# Retinal vasculopathy with cerebral leukoencephalopathy and systemic manifestations in conjunction with systemic lupus erythematosus: Missed diagnosis or misdiagnosis?

**DOI:** 10.1002/iid3.1367

**Published:** 2024-08-09

**Authors:** Xinhui Wang, Li Su, Jinming Han, Yilai Han, Yunsi Yin, Jiancheng Huang, Yi Tang, Yi Zhao, Qi Qin

**Affiliations:** ^1^ Department of Geriatrics, Henan Provincial People's Hospital People's Hospital of Zhengzhou University Zhengzhou China; ^2^ Innovation Center for Neurological Disorders, Department of Neurology Xuanwu Hospital, Capital Medical University, National Center for Neurological Disorders Beijing China; ^3^ Department of Rheumatology and Allergy Xuanwu Hospital, Capital Medical University Beijing China; ^4^ Department of Neurology Xuanwu Hospital, Capital Medical University Beijing China; ^5^ International School Capital Medical University Beijing China

**Keywords:** case report, corpus callosum atrophy, RVCL‐S, *TREX1* (three‐prime repair exonuclease‐1)

## Abstract

**Background:**

Retinal vasculopathy with cerebral leukoencephalopathy and systemic manifestations (RVCL‐S) is a rare autosomal dominant systemic microvascular disorder attributed to *TREX1* (three‐prime repair exonuclease‐1) gene mutations, often proned to misdiagnosed.

**Methods:**

We reported a case of RVCL‐S coexisting with systemic lupus erythematosus due to a mutation in the TREX1 gene. This study provided a summary and discussion of previously documented cases related to TREX1 mutations or RVCL‐S.

**Results:**

A 39‐year‐old female patient visited the clinic due to progressive memory loss and speech difficulties. Magnetic resonance imaging results showed corpus callosum atrophy and multiple subcortical calcifications in both brain hemispheres. Genetic testing revealed a TREX1 gene mutation (c.294dupA). Treatment with immunosuppressive therapy for 2 months led to improvements in communication and mobility. We also summarized previously reported cases providing an overview of *TREX*1 gene mutation or RCVL‐S.

**Conclusion:**

Our case establishes a compelling foundation for future RVCL‐S diagnosis and treatment paradigms. Notably, conducting systemic immunity screening in patients with RVCL‐S emerges as a strategic approach to prevent potential diagnostic oversights.

## Graphical abstract

Retinal vasculopathy with cerebral leukoencephalopathy and systemic manifestations (RVCL‐S) is a rare autosomal dominant systemic microvascular disorder attributed to TREX1 (three‐prime repair exonuclease‐1) gene mutations, which can be easily misdiagnosed. We present a case of RVCL‐S coexisting with systemic lupus erythematosus attributed to the TREX1 gene mutation.

## 1 INTRODUCTION

Retinal vasculopathy with cerebral leukoencephalopathy and systemic manifestations (RVCL‐S) is a rare autosomal dominant systemic microvascular disorder attributed to *TREX1* (three‐prime repair exonuclease‐1) gene mutations.^1^ This condition predominantly affects small blood vessels across various organs, prominently the eyes and brain.^2^ To date, RVCL‐S has been documented in 44 families,^3^ exhibiting a global distribution and unfortunately lacking a cure.^4^ In this case report, we present a distinctive case of RVCL‐S coinciding with systemic lupus erythematosus (SLE) that exhibited improvement following immunosuppressive therapy.

## 2 CASE PRESENTATION

A 39‐year‐old woman sought medical attention at the clinic, presenting a gradual onset of memory loss and speech fluency issues persisting over a span of 3 years. Her symptoms included disfluent speech, word‐finding impediments, auditory hallucinations, and mood fluctuations. Gait instability and diminished visual acuity had manifested over the preceding 8 months, coupled with photophobia and impaired vision, particularly in low‐light conditions, significantly impacting her daily activities. Noteworthy, her medical history was unremarkable, and consanguinity within her family lineage was absent. Neuropsychological assessments indicated a Mini‐Mental State Examination score of 14, a Montreal Cognitive Assessment score of 7, and a clinical dementia rating (CDR) of 1. Additionally, the Hamilton Depression Scale signified a depressive state.

Routine laboratory analyzes revealed leukopenia, decreased serum albumin levels, and increased thyroglobulin antibody titers. Immune‐related parameters, serum complement C3 and C4 levels, were significantly decreased, and elevated levels of rheumatoid factors were observed in the circulation. Positive results for anti‐nuclear antibody, anticardiolipin antibody, and lupus anticoagulant tests were documented. Cerebrospinal fluid (CSF) examination exhibited increased protein levels and normal cell counts, along with elevated concentrations of CSF IgA, IgM, IgG, and a 24‐h intrathecal IgG synthesis rate. The ratio of amyloid β‐protein (Aβ)1‐42/Aβ1‐40, t‐Tau, p‐Tau, antibody profiles of paraneoplastic syndromes, and antibody profile of autoimmune encephalitis were within normal limits in the CSF.

**Figure 1:**
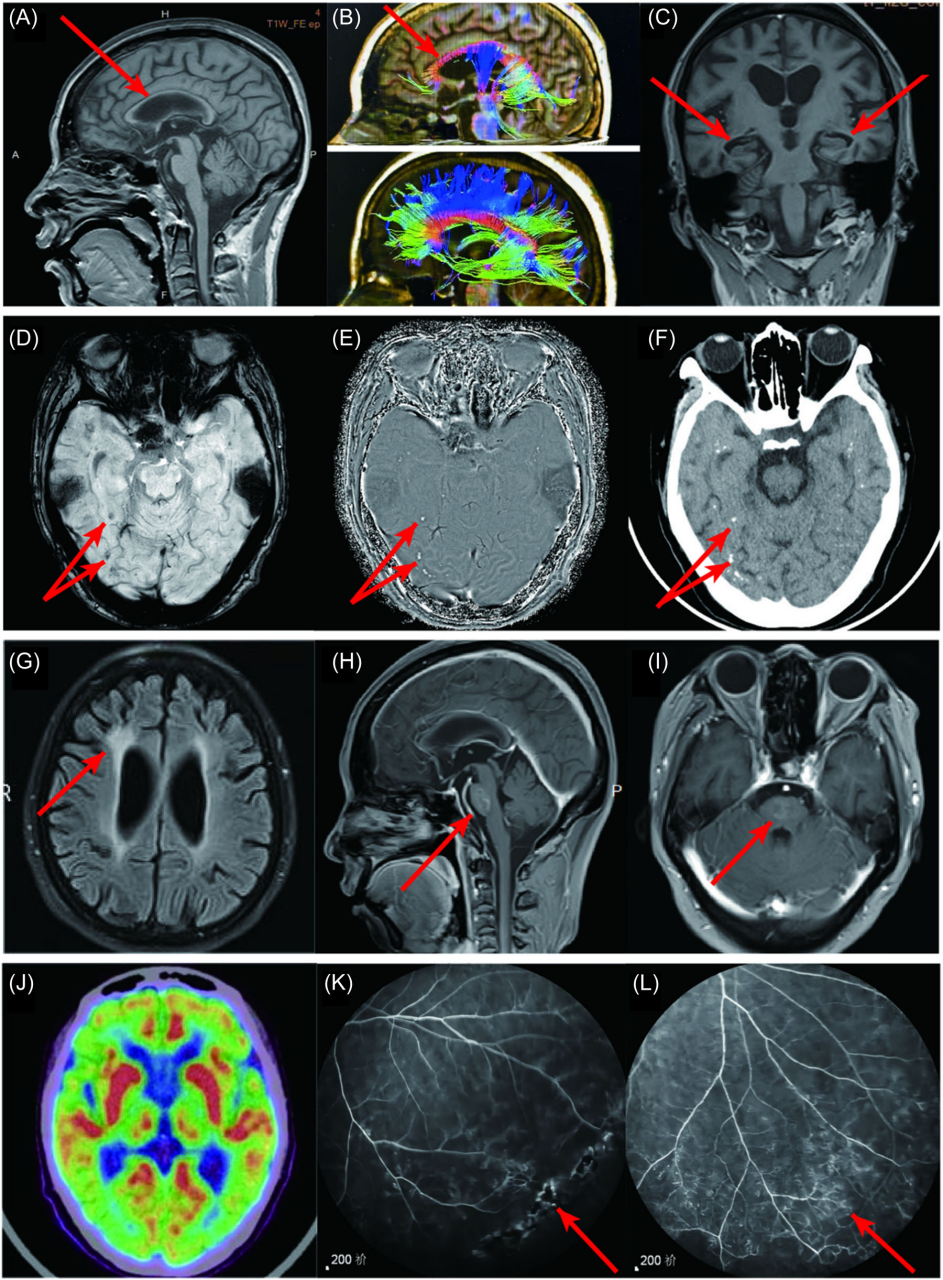
Neuroimaging features of the patient. (A) Sagittal brain magnetic resonance imaging (MRI) revealed corpus callosum atrophy. (B) Sagittal diffusion tensor imaging (DTI) showed a significantly reduced corpus callosum fiber tract in the patient (red arrow) compared to a normal healthy subject (bottom). (C) Coronal brain MRI demonstrated hippocampal atrophy (MTA2 grade). (D, E) Multiple subcortical spotty calcifications (red arrows) exhibited low signal during susceptibility weighted imaging (SWI) and high signal in the phase diagram. (F) Brain CT confirmed multiple subcortical punctate calcifications (red arrows) in the bilateral cerebral hemispheres. (G) Fluid‐attenuated inversion recovery (FLAIR) image exhibited high signal in the periventricular white matter of lateral ventricles. (H, I) Patchy enhancement was observed in the pons (red arrows). (J) 18F‐Fluorodeoxyglucose (18F‐FDG) positron emission tomography‐computed tomography (PET‐CT) indicated decreased metabolism in bilateral cerebral hemispheres. (K, L) Fluorescence fundus angiography (FFA) identified retinal microangiopathy in both eyes (red arrows).

Brain magnetic resonance imaging (MRI) displayed corpus callosum atrophy, coupled with numerous subcortical calcifications in bilateral cerebral hemispheres and accentuated patchy enhancements in the pons (Figure 1A–E). Brain computed tomography (CT) affirmed the presence of multiple subcortical punctate calcifications in bilateral cerebral hemispheres (Figure 1F). Moreover, 18F‐Fluorodeoxyglucose (18F‐FDG) positron emission tomography‐computed tomography (PET‐CT) findings indicated reduced metabolism in both cerebral hemispheres (Figure 1J). Corrected visual acuity was quantified as 0.6 (right) and 0.4 (left), respectively. Fluorescence fundus angiography delineated capillary dilation in the middle and peripheral retina, distinct regions of non‐perfusion, and microangiomas with intact arch rings in both eyes (Figure 1K,L).

Whole exome sequencing revealed numerous candidate single nucleotide variants, identified using GATK4 Best Practices and annotated according to American College of Medical Genetics and Genomics (ACMG) guidelines through multiple databases and tools (CADD, PolyPhen, and SIFT). Considering the proband's clinical symptoms, including multiple subcortical punctate calcifications, chilblain‐like skin lesions, and retinal degeneration, which are consistent with symptoms of TREX1 abnormalities, we believe that the mutation in TREX1 (c.294dupA) may be an important pathogenic factor. This mutation (c.294dupA) likely causes the TREX1 peptide chain to shorten from 314 to 202 amino acids.

## 3 DISCUSSION

The patient exhibited cognitive impairment, diminished vision, unsteady gait, and brain atrophy. Apart from corpus callosum atrophy on MRI, distinctive multiple subcortical calcifications in both cerebral hemispheres were observed—an atypical imaging finding not previously reported in neuropsychiatric lupus.^5^ Genetic analysis identified a heterozygous mutation in the TREX1 gene (c.294dupA). According to the guidelines of the ACMG, this mutation is classified as likely pathogenic, consistent with previous studies.^6^ The c.294dupA mutation may result in the production of a truncated protein at the carboxyl terminus (C‐terminal), reducing its length from 314 to 220 amino acids. Mutations in RVCL typically occur in the e C‐terminal domain of TREX1.^7^ The shortened carboxyl terminus may disrupt the region necessary for endoplasmic reticulum localization, leading to the loss of perinuclear localization of catalytically active TREX1. Sanger sequencing involving family members (parents and children) identified the same heterozygous TREX1 gene mutation in her mother, who had a history of frostbite and was suspected of microvascular disease. Subsequent in silico analysis supported the pathogenic nature of this mutation.

The TREX1 gene, encoding a 314‐amino acid protein, exhibits a complex phenotype‐genotype relationship, associated with human disorders such as Aicardi‐Goutieres syndrome, familial chilblain lupus, SLE, and RVCL‐S.^8^ A comprehensive review of over 40 patients with TREX1 gene mutations or RVCL‐S reported in the literature is presented in Table 1. The patient manifested typical presentations of RVCL‐S, including vascular retinopathy and brain dysfunction, while laboratory analysis supported the diagnosis of SLE. Patients with the diagnosis were based on the classification of SLE using standard EULAR/ACR‐2019,^22^ SLICC‐2012,^23^ the ACR‐1997,^24^ the SLEDAI‐2K^25^ and SLICC‐ACR.^26^ In terms of treatment, 12 patients underwent immunosuppressive therapy, predominantly corticosteroid therapy, resulting in improvement for 2 patients, partial improvement for 6 patients, and ineffectiveness for 4 patients. Most patients did not undergo systemic immunity screening, and only four previously reported cases were systematically tested for immune indicators. One case exhibited anti‐nuclear antibody (1:200) positivity with effective immunosuppressive therapy, while the remaining three patients had negative immunoassays, with two being unresponsive to immunosuppressive therapy. This suggests that patients without definite immune dysfunction may have a poor response to immunosuppressive therapy.

**Table 1:** Clinical and genetic characteristics of patients with *TREX1* mutation or RCVL‐S reported in literature.

Case number	Family member	Sex	Age at onset (years)	Migraine headache	Stroke‐like episodes	Cognitive impairment	Seizures	Psychiatric disease	Retinopathy	Renal insufficiency	Immune‐related parameters	Subcortical lesions on CT/MRI	*TREX1* mutation	Immunosuppressive therapy	Reference
1		F	36	+		+		+	+		SLE	+	C99fs	Improved	Presented case
2		F	38		+	+		+	+	+	−	+	D278fs	Partially improved	[9]
3		F	44	+	+				+		Unknown	+	R284fs		[10]
4		M	55		+	+			+	+	Unknown	+	T249fs	Partially improved	[11]
5		M	30	+	+				+	+	antinuclear antibodies (1:200)	+	T249fs	Improved	[12]
6		F	32				+		+		Unknown	+	R284fs		[13]
7		M	32	+	+			+	+		Unknown	+	T236fs	Partially improved	[14]
8		M	36	+	+		+	+	+	+	Unknown	+	T270fs	Partially improved	[15]
9		F	35	+			+	+	+	+	Unknown		V235fs	Ineffective	
10	III	F	44	+	+				+		Unknown	+	V235fs		[16]
11	II	M	43	+	+							+			
12	II‐1	F	41		+	+			+		Unknown	+	V235fs		[9, 17]
13	II‐4	M	50	+		+	+			+	Unknown	+		Partially improved	
14	III‐4	F	30	+	+	+	+	+	+	+	−	+		Ineffective	
15	Brother	F	35	+	+	+			+		Unknown	+	V235fs	Partially improved	[18]
16		F	41	+	+	+	+	+				+			
17		M	44						+			−	V235fs		[9, 19]
18		M	54					+	+			+			
19		F	54	+		+						+			
20		M	56				+		+			+			
21		M	43	+	+				+			+			
22		M	41	+	+				+			+			
23		M	56		+	+			+			+		Ineffective	
24	I‐1	F	50	+	+	+			+		Unknown	Unknown	T249fs	Improved	[9, 20]
25	II‐2	F	39	+	+	+			+			+			
26	II‐5	F	43	+	+	+			+			Unknown			
27	III‐2	F	31	+	+	+			+	+		+			
28	III‐3	M	32		+	+		+	+			+			
29	III‐6	M	36	+	+	+			+			+			
30	III‐9	M	Known	+					+	+		+			
31	III‐10	F	40		+	+		+	+			+			
32	III‐11	M	42		+	+		+	+			+			
33	III‐14	F	39		+	+			+			+			
34	III‐17	M	40	+	+	+		+	+	+		Unknown			
35		F	50	+	+	+			+		−	+	V235fs	Ineffective	[3]
36‐45	A Dutch family，n = 11			+					+		Unknown	+	V235fs		[2, 9, 21]

TREX1, primarily localized to white matter Iba1+ microglia associated with microvasculature adjacent to ischemic lesions, suggests a role for TREX1 in responding to ischemia.^27^ TREX1 mutations associated with RVCL‐S involve the C‐terminal frameshift mutations, which prevent proper localization of TREX1 to the endoplasmic reticulum. This mis‐localization disrupts normal vascular endothelial functions, leading to vasculopathy, which is a hallmark of RVCL‐S. In contrast, the role of TREX1 in SLE primarily involves the enzyme's exonuclease activity and the prevention of excessive immune activation through the cGAS‐STING pathway. The patient showed improvements in specific symptoms that are characteristic of RVCL‐S, such as retinal vasculopathy and cerebral white matter changes. These symptoms are distinct from those typically seen in SLE and are directly linked to the vascular endothelial damage caused by TREX1 C‐terminal mutations. Microangiopathy resulting from TREX‐1 gene mutation forms the basis of RVCL‐S complicated with SLE in this patient, culminating in a diagnosis of ‘RVCL‐S coexisting with SLE’. Ophthalmic consultation considered a clinical diagnosis of retinal vasculitis and uveitis due to pronounced vision impairment and retinal microangiopathy.

The uvea, comprising the iris, ciliary body, and choroid, may serve as a target of autoimmunity.^28^ Compromised iris function contributes to photophobia and impaired light reflexes, while choroid disruption impairs light reception and transmission, resulting in reduced sensitivity to dimly lit environments. The patient underwent laser coagulation of retinal lesions. Following a 2‐month therapeutic regimen, including methylprednisolone, mycophenolate mofetil, hydroxychloroquine, and tandospirone, the patient had good medication compliance; improvements were noted in communication and ambulation functions. The overall cognitive assessment CDR score remained at 1, while corrected visual acuity measured 0.5 (right) and 0.4 (left), respectively.

Despite the absence of a specific treatment for RVCL‐S, immunosuppressants may represent a potential avenue for alleviating symptoms and improving patients' quality of life, underscoring the intriguing coexistence of RVCL‐S and abnormal immune function associated with the heterozygous *TREX1* mutation.

## 4 CONCLUSION

This case establishes a compelling foundation for future RVCL‐S diagnosis and treatment paradigms. Notably, conducting systemic immunity screening in RVCL‐S patients emerges as a strategic approach to prevent potential diagnostic oversights. Given the potential adverse reactions of immunosuppressive drugs, we advocate for immunosuppressive therapy in patients presenting with abnormal immune function. Embracing a multidisciplinary approach has the potential to catalyze innovative insights for the management of rare neurological disorders.

## AUTHOR CONTRIBUTIONS

**Xinhui Wang:** Formal analysis; investigation; data curation; writing—original draft. **Li Su:** conceptualization; investigation. **Jinming Han:** Visualization; writing—review and editing. **Yilai Han and Yunsi Yin:** technical assistance. **Jiancheng Huang:** Writing—review and editing. **Yi Tang:** Resources; project administration. **Yi Zhao:** Methodology. **Qi Qin:** Resources; supervision; writing—review and editing.

## CONFLICT OF INTEREST STATEMENT

The authors declare no conflicts of interest.

## ETHICS STATEMENT

The patient consented to the publishing of this case report.

## Data Availability

The data that support the findings of this study are available on request from the corresponding author. The data are not publicly available due to privacy or ethical restrictions.
